# Beneficial Effects of an Aged Black Garlic Extract in the Metabolic and Vascular Alterations Induced by a High Fat/Sucrose Diet in Male Rats

**DOI:** 10.3390/nu11010153

**Published:** 2019-01-12

**Authors:** Sara Amor, Daniel González-Hedström, Beatriz Martín-Carro, Antonio Manuel Inarejos-García, Paula Almodóvar, Marin Prodanov, Angel Luis García-Villalón, Miriam Granado

**Affiliations:** 1 Departamento de Fisiología, Facultad de Medicina, Universidad Autónoma de Madrid. C/Arzobispo Morcillo n°2 28029 Madrid, Spain; sara.amor@uam.es (S.A.); dgonzalez@pharmactive.eu (D.G.-H.); beatriz.martinc@uam.es (B.M.-C.); angeluis.villalon@uam.es (A.L.G.-V.); 2 Pharmactive Biotech Products SL, Parque Científico de Madrid, 28049 Madrid, Spain; aminarejos@pharmactive.eu (A.M.I.-G.); palmodovar@pharmactive.eu (P.A.); 3 Departamento de Química Física Aplicada, Facultad de Ciencias, CIAL (CEI, CSIC-UAM), Universidad Autónoma de Madrid, 28049 Madrid, Spain; marin.prodanov@uam.es; 4 CIBER Fisiopatología de la Obesidad y Nutrición. Instituto de Salud Carlos III, 28029 Madrid, Spain

**Keywords:** aged black garlic, metabolic syndrome, obesity, adipose tissue, insulin, cardiovascular, rat

## Abstract

Aged black garlic (ABG) is a functional food with antioxidant and anti-inflammatory properties. Recent studies also report its beneficial metabolic effects in a context of obesity or diabetes, although the mechanisms involved are poorly understood. The aim of this work was to analyze the effects of an ABG extract in the vascular and metabolic alterations induced by a high-fat/sucrose diet in rats. For this purpose, male Sprague–Dawley rats were fed either a standard chow (controls; *n* = 12) or a high-fat/sucrose diet (HFD; *n* = 24) for 16 weeks. From week 8 on, half of the HFD rats were treated with a commercial ABG extract concentrated in S-allyl cysteine and melanoidins (ABG10+®; 250 mg/kg daily by gavage; 5 mL/kg). ABG10+®-treated rats showed lower mean caloric intake, body weight, triglycerides, low density lipoprotein cholesterol (LDL-c), insulin and leptin serum concentrations and higher high density lipoprotein cholesterol (HDL-c) and adiponectin serum concentrations than non-treated rats. In the hypothalamus, ABG10+® treatment induced an increase in the gene expression of proopiomelanocortin (*POMC*) and a decrease in leptin receptor (*ObR*) mRNA levels. No significant changes were found in visceral adipose tissue except for an overexpression of β3-adrenergic receptor (*β3-ADR*) in ABG-treated rats. In subcutaneous adipose tissue, ABG10+® treatment decreased adipose weight and downregulated the gene expression of *PPAR-γ*, *LPL*, *ObR* and *HSL*. In brown adipose tissue, an overexpression of *InsR*, *GLUT-4*, *UCP-1* and *β3-ADR* in ABG10+®-treated rats was found, whereas PPAR-γ mRNA levels were significantly decreased. Regarding vascular function, ABG10+® treatment attenuated the obesity-induced vasoconstriction in response to potassium chloride both in presence/absence of perivascular adipose tissue (PVAT). On the contrary, aorta segments from ABG-treated rats showed and improved relaxation in response to acetylcholine only when PVAT was present, with this fact possible being related to the decreased gene expression of proinflammatory cytokines in this tissue. In conclusion, ABG10+® administration partially improves the metabolic and vascular alterations induced by a high-fat/high-sucrose diet in rats through modifications in the gene expression of proteins and neuropeptides involved in inflammation, fat metabolism and food intake regulation. Further studies are required to assess the bioavailability of ABG between rats and humans.

## 1. Introduction

In recent decades, increased life expectancy, sedentary lifestyle and consumption of foods rich in saturated fats and added sugars have exposed the population of the developed countries to emerging health problems. Among them, the steadily increasing incidence of obesity and associated morbidities is recognized as a major public health problem, reaching epidemics proportions in both industrialized and developing countries.

In obesity, adipose tissue depots are subjected to extensive hypertrophy and to the secretion of adipokines and proinflammatory cytokines by both adipocytes and infiltrating immune cells [1], which derives in a condition of low-grade inflammation and in the development of insulin resistance in the long term [2]. 

In the past few years, numerous drugs have been approved for the treatment of obesity and its related co-morbidities. However, most of them have been withdrawn from the market because of their adverse effects [3]. For this reason, finding a new drug with no side effects is the ultimate goal for many pharmaceutical companies. 

Among natural phytochemical products, several studies have reported beneficial effects of aged black garlic (ABG) preventing some of the cardiometabolic alterations associated to metabolic syndrome, both in humans and in experimental animals [4,5]. 

ABG is produced through the application of heat (60–90 °C) and controlled humidity (80–90%) to raw garlic bulbs during short periods of time that vary depending on cultures, manufacturers and purposes. During the aging process of black garlic, the Maillard reaction takes place, originating the typical dark brown color and conferring a jelly-like texture and a sweet and sour taste to the final product [4]. By this process, most of the molecules responsible for the harsh and irritating flavor typical of fresh raw garlic (i.e., allicin) are oxidized and/or converted into more stable compounds such S-allyl cysteine (SAC) and S-allylmercaptocysteine (SAMC), which are recognized as potent antioxidants [5]. Indeed, SAC has been proposed as the most appropriate quality marker of ABG [6]. Other macromolecules obtained during the aging process are melanoidins, compounds responsible for the dark color in several foods such as coffee, cocoa, beer, and honey. [7]. Melanoidins have been studied, not only because of their nutritional value, but also because of their anti-inflammatory and antioxidant capacity [8], as they exert a radical-scavenging activity with beneficial effects on the lipid profile [9,10]. 

In addition to its antioxidant properties, ABG is reported to exert other biological functions which include anti-inflammatory [11], neuroprotective [12], anti-neoplasic [13] anti-obesity [14], anti-diabetic [15], anti-allergic [16], cardioprotective [17], and hepatoprotective [18] effects, among others. 

Regarding its anti-diabetic and anti-obesity effects, some studies have been published on the beneficial role of ABG improving the lipid profile and insulin sensitization in an obesity and/or diabetes context, both in experimental animals [19,20,21] and in humans [22]. However, the mechanisms by which ABG exerts these effects are poorly understood. Thus, the aim of this work was to study the effects of ABG treatment in the metabolic and vascular alterations induced by a high-fat/high-sucrose diet by analyzing the gene expression of different markers related to energy homeostasis in the hypothalamus and in different adipose tissue depots.

## 2. Material and Methods

### 2.1. Materials

Five samples of ABG extracts marketed under the brand name ABG10+® were provided by Pharmactive Biotech Products S.L. All of them were in a powder form and were stored in darkness until analysis was performed. 

SAC and alliin were purchased from Sigma-Aldrich (Madrid, Spain); heptanesulfonic acid sodium salt and orthophosphoric acid were purchased from Fisher Scientific (Santa Clara, CA, USA). HPLC solvents were from Merck (VWR International, Llinars del Vallès, Spain). Ultrapure water for HPLC was obtained from a Milli-Q system (Millipore Corp., Bedford, MA, USA).

### 2.2. Physical-Chemical Characterization of ABG10+® Extracts

#### 2.2.1. Absorption Spectra of ABG10+®

The ultraviolet-visible (UV-Vis) spectra of ABG10+® were obtained according to Kang et al [23]. Briefly, 1 mg/mL of ABG10+® solution was prepared in water and filtered through a 0.45 μm Nylon membrane filter. Different sample dilutions were prepared in order to obtain the UV-Vis spectra of the ABG10+® solution. The absorption spectra of the samples were acquired by scanning them from 200 to 500 nm by the use of a Beckman Coulter DU® Series 730 Life Science UV/Vis Scanning Spectrophotometer (Indianapolis, IN, USA). 

#### 2.2.2. Analysis of Organosulfur Aminoacids by HPLC-PAD/MSD

Quantification of SAC in ABG10+® samples was carried out by High Performance Liquid Chromatography coupled to an electrospray ionization (ESI) mass spectrometry detector (MSD), according to Bae et al. [6]. An Agilent Technologies 1220 Infinity series HPLC system, (Palo Alto, CA, USA) and a C18-PFP Ultra-Inert HPLC Column (250 × 4.6 mm, 5 µm particle size) (ACE, Scotland) were used.

Identification and quantification of the organosulfur compounds were also carried out by HPLC coupled to an electrospray ionization mass spectrometry (ESI-MS) detector, according to the method proposed by García-Villalón et al. [17]. Briefly, the chromatographic system was an Agilent 1200 series, (binary pump, autosampler, photodiode array detector (PAD)), coupled to an ESI source and quadrupole mass analyzer. The stationary phase (Luna C18-PFP column (250 × 4.6 mm and 5 μm; 100 Å)) from Phenomenex, S.L. (Madrid, Spain) was termostatized at 25 °C. The mobile phase was pumped at a flow rate of 0.5 mL/min and was a linear gradient of component A (0.1% (*v*/*v*) formic acid in water), and component B (acetonitrile) as follows: from 0 to 20 min, 10% to 90% of B; during 10 min, 90% of B; from 90% to 10% of B in 1 min and 10 min at 90% B for conditioning the column for the next analysis. The PAD was set at 208 nm and the injection volume was 20 μL. The total run time was 41 min. ESI-MS was tuned as follows: mass range (SCAN): from 50 to 1500 umas, ionization mode: ESI+, drying gas flow: 9 L/min, nebulizer pressure: 60 psig, drying gas temperature: 250 °C, vaporizer temperature: 150 °C, capillary tension: 2000 V, charging tension: 2000 V, and fragmentator tension: 40 V. 

The organosulfur aminoacids were quantified by external calibration curves according to their chemical structure, molecules with the sulfoxide group were quantified as alliin, and the non-sulfoxide molecules were quantified as SAC. 

### 2.3. In Vivo Experiments

#### 2.3.1 Animals

All the experiments were conducted according to the European Union legislation and with the approval of the Animal Care and Use Committee of the Community of Madrid (Spain).

Twenty-four 3-months-old male Sprague–Dawley rats were housed three per cage and maintained in climate-controlled quarters with a 12 h light cycle and under controlled conditions of humidity (50–60%) and temperature (22–24 °C). Rats were fed ad libitum either a standard chow (controls; *n* = 12) or a high-fat diet (test diets, Cat. #58V8) containing 45% energy from fat and 5% sucrose added to the drinking water for 12 weeks (HFD; *n* = 24). Half of the rats were treated with an ABG extract ABG10+® (250 mg/kg; *n* = 12) orally during their last month of life and the other half were treated with vehicle (5 mL/kg). A weekly control of body weight and solid and liquid intake was performed. All animals were sacrificed at 6 months of age by decapitation after an intraperitoneal injection of sodium pentobarbital (100 mg/kg). The night before sacrifice, all animals were subjected to 12 h of fasting. 

After decapitation, blood was collected and centrifuged at 3000 rpm for 20 min to obtain the serum, which was kept frozen at −80 °C until further analysis. In addition, epididymal visceral, lumbar subcutaneous, brown and periaortic adipose tissue, hypothalamus, kidneys, adrenal glands, spleen, liver, soleous and gastrocnemius muscle were immediately removed and weighed. Subsequently, all tissues were stored at −80 °C for later analysis.

#### 2.3.2. Oral Glucose Tolerance Test (OGTT) and Homeostatic Model Assessment of Insulin Resistance (HOMA-IR)

One week before sacrifice, all rats were subjected to an OGTT. For this purpose, they were fasted overnight and the following morning the basal glycaemia was measured by venous tail puncture using Glucosard^TM^ G (Arkray Factory, Inc., Koji Konan-cho, Koka, Shiga, Japan). Rats were then orally administered a bolus of glucose (3 mg/g bw) and glycaemia was measured 30, 60 and 120 min afterwards. The total area under the curve (AUC) for the glucose response was calculated with the following formula: AUC = 25 × (fasting value) + 0.5 × (30 min value) + 0.75 × (1 h value) + 0.5 × (2 h value) [24]. The HOMA-IR index was calculated through the following formula: fasting glucose (mg/dL) × (fasting insulin (ng/m)/405 [25].

#### 2.3.3. Serums Measurements

(a) Metabolic Hormones

Serum concentrations of leptin, insulin, and adiponectin were measured by ELISA following the manufacturer’s instructions (Merck Millipore, Dramstadt, Germany). Absorbance was measured in each well using a BioTek Synergy HT spectrometer (Winooski, VT, USA). The sensitivity of the method for leptin, insulin, and adiponectin was 0.04, 0.2, and 0.16 ng/mL, respectively. The intra-assay variation was between 1.9–2.5% for leptin, 0.9–8.4% for insulin, and 0.43–1.96% for adiponectin.

(b) Lipid Profile

Triglycerides, total cholesterol, low-density lipoprotein (LDL), and high-density lipoprotein (HDL) were measured in the serum using commercial kits from Spin React S.A.U (Sant Esteve de Bas, Gerona, Spain) and following the manufacturer’s instructions. 

#### 2.3.4. RNA Extraction and Quantitative Real-Time PCR

Total RNA was extracted from the hypothalamus, visceral, subcutaneous and brown adipose tissue according to the Tri-Reagent protocol [26]. cDNA was then synthesized from 1 µg of total RNA using a high-capacity cDNA RT kit (Applied Biosystems, Foster City, CA, USA).

Neuropeptide Y (NPY), agouti-related protein (AgRP), proopiomelanocortin (POMC), cocaine amphetamine-related transcript (CART), insulin receptor (InsR) and leptin receptor (ObR) mRNAs were measured in hypothalamic samples by real-time PCR. In visceral, subcutaneous, and perivascular adipose tissue (PVAT) the gene expression of *InsR*, *ObR*, fatty acid synthetase (*FASN*), hormone-sensitive lipase (*HSL*), lipoprotein lipase (*LPL*), peroxisome proliferator-activator receptor-γ (*PPAR-γ*), glucose transporter type 4 (*GLUT-4*), beta-3 adrenergic receptor (*β3-ADR*), interleukin-1 beta (*IL-1β*), interleukin-6 (*IL-6*), tumor necrosis factor-alpha (*TNF-α*), inducible nitric oxide synthase (*iNOS*) and NADPH oxidase-4 (*NOX-4*) were analyzed. Finally, in brown adipose tissue, the mRNA concentrations of InsR, ObR, FASN, HSL, LPL, PPAR-γ, GLUT-4, β3-ADR, carnitine palmitoyltransferase-I (CPT-1) and uncoupling protein 1 (UCP-1) mRNAs were assessed, by using assay on-demand kits (Applied Biosystems) for each gene. TaqMan Universal PCR Master Mix (Applied Biosystems) was used for amplification according to the manufacturer’s protocol in a Step One System (Applied Biosystems). Values were normalized to the housekeeping 18S. To determine relative expression levels, the ∆∆C_T_ method was used [27]. Statistics were performed using ∆∆C_T_ values.

#### 2.3.5. Vascular Reactivity Experiments

Immediately after sacrifice, the thoracic aorta was carefully dissected, cut in 2 mm segments and kept in a cold isotonic saline solution. Half of the segments were completely denuded from PVAT, whereas the other half maintained the PVAT in order to study their vascular reactivity in the most similar conditions possible to those of the in vivo model. Each aorta segment was set in a 4 mL organ bath containing modified Krebs–Henseleit solution at 37 °C (mM): NaCl, 115; KCl, 4.6; KH_2_PO_4_, 1.2; MgSO_4_, 1.2; CaCl_2_, 2.5; NaHCO_3_, 25; glucose, 11. The solution was equilibrated with 95% oxygen and 5% carbon dioxide to a pH of 7.3–7.4. Briefly, two fine steel wires (100 μm of diameter) were passed through the lumen of the vascular segment. One wire was fixed to the organ bath wall and the other wire was connected to a strain gauge for isometric tension recording (Universal Transducing Cell UC3 and Statham Microscale Accessory UL5, Statham Instruments, Inc.). This arrangement permits application of passive tension in a perpendicular plane to the long axis of the vascular cylinder. The changes in isometric force were recorded using a PowerLab data acquisition system (ADInstruments, Colorado Springs, CO, USA). After applying an optimal passive tension of 1 g, vascular segments were allowed to equilibrate for 60–90 min. After equilibration, all segments were stimulated with potassium chloride (KCl; 100 mM) to determine the contractility of smooth muscle. Afterwards, a single dose of acetylcholine (10^−6^ M) was added to each aorta segment previously pre-contracted with phenylephrine 10^−7.5^ M (Sigma-Aldrich, St. Louis, MO, USA). The relaxation in response to acetylcholine was determined based on the percentage of the active tone achieved by the NO donor sodium nitroprusside (10^−5^ M) (Sigma-Aldrich, St. Louis, MO, USA). 

### 2.4. Statistical Analysis

The statistical analysis was performed with the software Prisma GraphPad 5.0. (San Diego, CA, USA). All data are represented as mean ± standard error mean (SEM). Data of body weight, food and water intake were analyzed by repeated measures two-way ANOVA. Differences in organ weights were also assessed by analysis of covariance (ANCOVA) to determine if they are explained by variations in body weight. The rest of the data were analyzed by one-way ANOVA followed by the Bonferroni post-hoc test. Differences were considered significant when *p* < 0.05.

## 3. Results

### 3.1. Physicochemical Characterization of ABG10+® Extract

**Figure 1:**
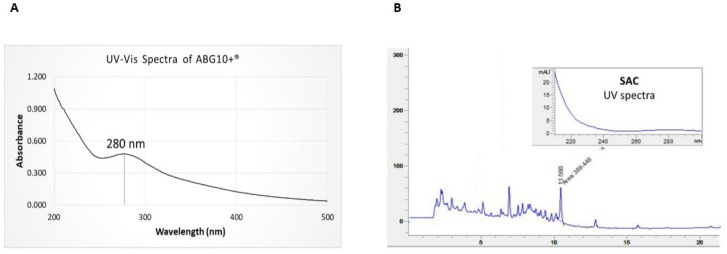
UV-Vis spectra of ABG10+® in water solution (**A**) and HPLC-PAD chromatogram of the S-allyl cysteine (Retention time (R_T_): 11.58 min) in ABG10+® (**B**).

The UV-Vis spectra of the ABG10+® extract dissolved in water at a concentration of 0.5 mg/mL showed a characteristic absorbance peak at 280 nm (Figure 1A). The maximum absorbance at 280 nm was explained by the presence of phenolic compounds and aminoacids which are involved in the early Maillard reaction during ageing process, obtaining initial intermediates arising from sugar-amine condensation and Amadori rearrangement. 

The chromatogram of the ABG10+® extract obtained from the HPLC-MS analysis is shown in Figure 1B. The retention time (RT) of SAC was 11.5 min, and its concentration in ABG10+® employed in the in vivo trial was over 0.1% (0.12 ± 0.01%, dry basis).

Bioactive components of ABG10+® were also identified and quantified by HPLC-MS (Table 1). Among the organosulfur components in ABG10+®, the most concentrated was SAC for RT of 7.4 min (1.15 ± 0.02 mg/g), together with *iso*-SAC for RT of 8.3 min (0.04 ± 0.00 mg/g), followed by alliin for RT of 5.6 min (0.03 ± 0.00 mg/g) and *iso*-alliin for RT of 5.2 min (0.01 ± 0.00 mg/g).

### 3.2. In Vivo Experiments in Rats

#### 3.2.1. Body and Organ Weights

**Figure 2:**
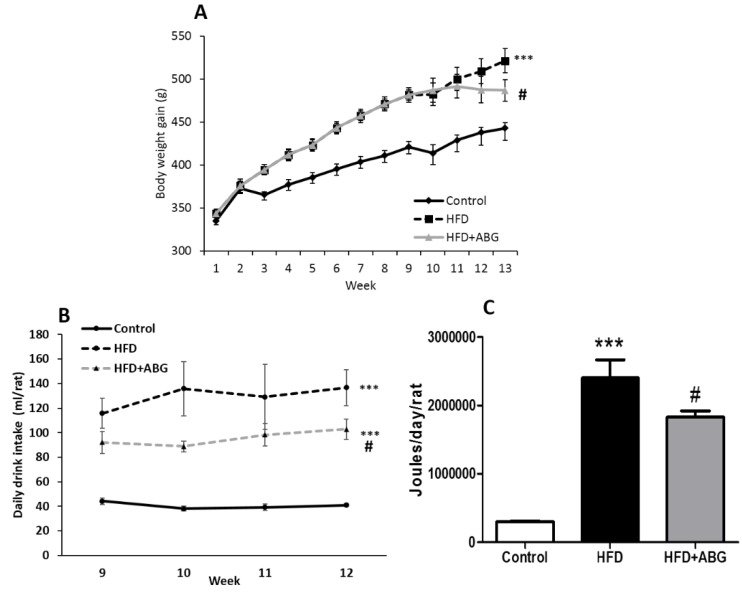
Body weight gain (**A**), mean daily drink intake (**B**), and mean daily caloric intake (**C**) of rats fed a standard chow (control) or a high-fat/sucrose diet (HFD) and treated with vehicles or HFD + ABG. Data are represented as mean value ± SEM; *n* = 12 rats/group. *** *p* < 0.01 vs. control; ^#^ *p* < 0.05 vs. HFD.

Body weight gain is shown in Figure 2A. The two-way ANOVA analysis showed a significant interaction between the two factors (time and experimental group) (F = 10.85; *p* < 0.001), and a significant effect of both time (F = 421.6; *p* < 0.001) and experimental group (F = 9.095; *p* < 0.001). The post-hoc analysis revealed a significant increase in body weight gain in HFD-fed rats compared to controls (*p* < 0.001). ABG treatment for one month (weeks: 9–12) significantly decreased body weight gain in rats fed an HFD (*p* < 0.05). 

Weights of organs and tissues are shown in Table 2. Using body weight as a covariate, we found that weights of heart, kidneys, adrenal glands, spleen, liver, soleous and gastrocnemius muscle did not show differences between controls, HDF and HDF rats treated with ABG after removing the effect of body weight. However, epididymal (*p* < 0.0001), subcutaneous (*p* < 0.0001), brown (*p* < 0.0002) and perivascular (*p* < 0.004) adipose tissue depots were increased by HFD after removing the effect of body weight. ABG-treated rats showed decreased subcutaneous (*p* < 0.02) and brown (*p* < 0.0001) adipose tissues compared to non-treated HFD rats.

#### 3.2.2. Daily Caloric Intake

The mean daily caloric intake was significantly increased in all HFD rats compared to controls (Figure 2C; *p* < 0.001). No statistical differences were found in the daily food intake between HFD-treated and non-treated rats (data not shown). However, both the daily drinking intake of water supplemented with 5% sucrose (Figure 2B) and the daily mean caloric intake were significantly lower in ABG-treated rats compared to non-treated rats (*p* < 0.05 for both).

#### 3.2.3. Triglycerides, Total Cholesterol, LDL Cholesterol and HDL Cholesterol

Triglycerides, total cholesterol, LDL cholesterol and HDL cholesterol serum concentrations are shown in Table 2. Compared to controls, HFD rats showed increased serum levels of triglycerides (*p* < 0.01), total cholesterol (*p* < 0.05) and LDL cholesterol (*p* < 0.05) whereas the circulating levels of HDL cholesterol were significantly reduced (*p* < 0.01). ABG treatment did not modify the serum concentrations of total cholesterol but it significantly decreased LDL cholesterol and triglycerides (*p* < 0.05 for both) and increased HDL cholesterol (*p* < 0.01).

#### 3.2.4. Glycaemia, Insulin, Leptin and Adiponectin Serum Concentrations

No significant differences were found in basal glycaemia among experimental groups (Table 2). HFD rats showed increased serum concentrations of leptin (*p* < 0.01; Table 2) and insulin (*p* < 0.05; Table 2) compared to controls. ABG treatment prevented the obesity-induced increase in the circulating levels of these two hormones and significantly increased adiponectin (*p* < 0.05; Table 2) serum levels.

#### 3.2.5. Oral Glucose Tolerance Test (OGTT) and HOMA Index

**Figure 3:**
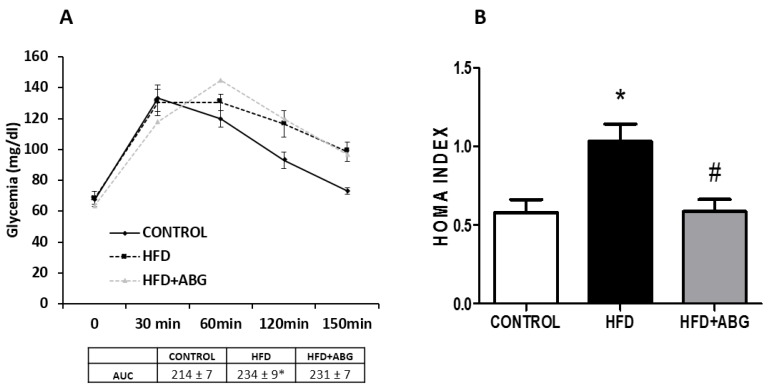
Oral glucose tolerance test, area under the curve (**A**) and HOMA index (**B**) of rats fed a standard chow (control) or a high-fat diet (HFD)/high-sucrose diet and treated with vehicles or HFD + ABG. Data are represented as mean ± SEM; *n* = 8 samples/group. * *p* < 0.05 vs. control; ^#^ *p* < 0.05 vs. HFD).

Results of OGTT are shown in Figure 3A. The two-way ANOVA analysis showed a significant interaction between the two factors (experimental group and time) (F = 3.809; *p* < 0.01) and a significant effect of the factor “time” (F = 62.7; *p* < 0.001). The post-hoc analysis revealed no significant changes between experimental groups in basal glycemia. An oral glucose bolus of 3 g/kg induced, 30 min later, a significant increase in glycaemia in all experimental groups with this increase being statistically lower in ABG-treated rats (*p* < 0.05). Glycaemia levels were maintained elevated 60 min after glucose administration in all experimental groups with no significant differences among them. One hundred and fifty minutes after the oral bolus of glucose control, rats recovered their basal glucose levels whereas glycaemia remained elevated both in HFD and in HFD + ABG rats. The area under the curve was significantly increased in HFD rats, regardless of the treatment that had been received (*p* < 0.05). However, ABG treatment significantly reduced the obesity-induced increase in HOMA index (Figure 3B; *p* < 0.05).

#### 3.2.6. NPY, AgRP, POMC, CART, InsR, ObR mRNA, TNF-α and IL-1β Levels in the Hypothalamus

**Figure 4:**
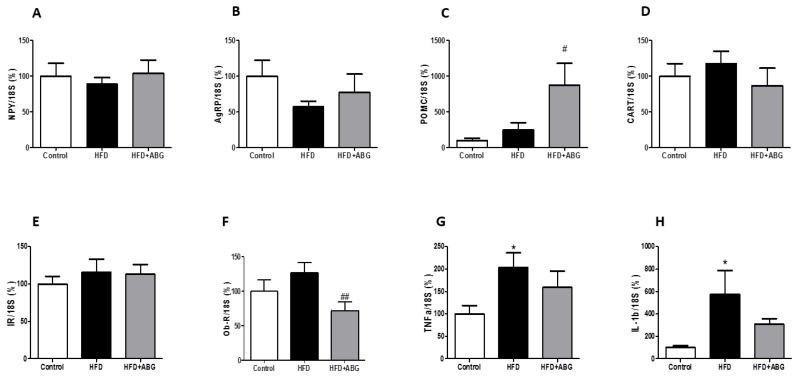
mRNA concentrations of neuropeptide Y (**A**), agouti-related protein (**B**), proopiomelanocortin (**C**), cocaine and amphetamine related transcript (**D**), insulin receptor (**E**), leptin receptor (**F**), TNF-α (**G**) and IL-1β (**H**) in the hypothalamus of rats fed a standard chow (control) or a high-fat diet (HFD)/high-sucrose diet and treated with vehicles or HFD + ABG. Data are represented as mean ± SEM; *n* = 8 samples/group. * *p* < 0.05 vs. control; ^#^ *p* < 0.05 vs. HFD; ^##^ *p* < 0.01 vs. HFD.

Figure 4A–H shows NPY, AgRP, POMC, CART, InsR, ObR, TNF-α and IL-1β mRNA levels, respectively, in the hypothalamus. HFD rats showed no differences in the gene expression of *NPY*, *AgRP*, *POMC*, *CART*, *InsR* and *ObR*, but they showed a significant upregulation of TNF-α and IL-1β compared to controls (*p* < 0.05). The mRNA levels of NPY, AgRP, CARTand InsR were unchanged between HFD and HFD + ABG rats. However, ABG treatment induced a significant upregulation in the mRNA levels of POMC (*p* < 0.01) and a down regulation in the gene expression of *ObR* in the hypothalamus (*p* < 0.05). The mRNA levels of TNF-α and IL-1β in HFD + ABG rats were not statistically different, neither from HFD nor from control rats. 

#### 3.2.7. FASN, HSL, LPL, PPAR-γ, ObR, InsR, GLUT-4, β3-ADR, IL-1β, IL-6, TNF-α and NOX-4 mRNA Levels in Visceral and Subcutaneous Adipose Tissue 

In visceral epididymal adipose tissue, no differences were observed between experimental groups, neither in the gene expression of pro-oxidant and pro-inflammatory markers nor in the mRNA levels of metabolic proteins such as FASN, HSL, LPL, PPAR-γ, ObR, InsR and GLUT-4 (data not shown). However, the gene expression of the *β3-ADR* was strongly upregulated in HFD + ABG rats compared to HFD rats (775 ± 350 vs. 100 ± 27; *p* < 0.001). 

**Figure 5:**
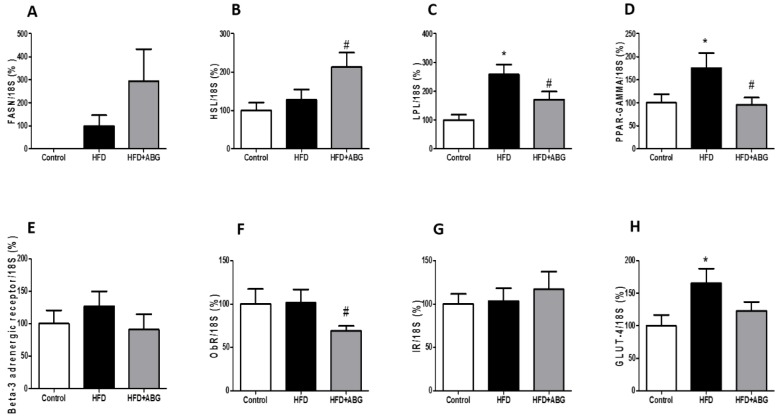
mRNA concentrations of fatty acid synthetase (**A**), hormone-sensitive lipase (**B**), lipoprotein lipase (**C**), peroxisome proliferator-activated receptor gamma (**D**), beta-3 adrenergic receptor (**E**), leptin receptor (**F**), insulin receptor (**G**) and glucose transporter 4 (**H**) in subcutaneous adipose tissue of rats fed a standard chow (control) or a high fat diet/sucrose diet (HFD) and treated with vehicles or HFD + ABG. Data are represented as mean ± SEM; *n* = 8 samples/group. * *p* < 0.05 vs. control; ^#^ *p* < 0.05 vs. HFD.

In subcutaneous lumbar adipose tissue, HFD rats showed increased mRNA levels of LPL (*p* < 0.05; Figure 5C), PPAR-γ (*p* < 0.05; Figure 5C) and GLUT-4 (*p* < 0.05; Figure 5H) compared to controls. ABG treatment did not modify the gene expression of *FASN*, *InsR*, *β-ADR* and *GLUT-4* but it significantly upregulated the gene expression of *HSL* (*p* < 0.05; Figure 5B) and downregulated the mRNA levels of LPL (*p* < 0.05; Figure 5C), PPAR-γ (*p* < 0.05; Figure 5D) and ObR (*p* < 0.05; Figure 5F). No statistical differences were found in the gene expression of pro-oxidant and pro-inflammatory markers among the three experimental groups (data not shown).

#### 3.2.8. FASN, HSL, LPL, PPAR-γ, β3-ADR, ObR, InsR, GLUT-4, CPT-1 and UCP-1 mRNA Levels in Brown Adipose Tissue

**Figure 6:**
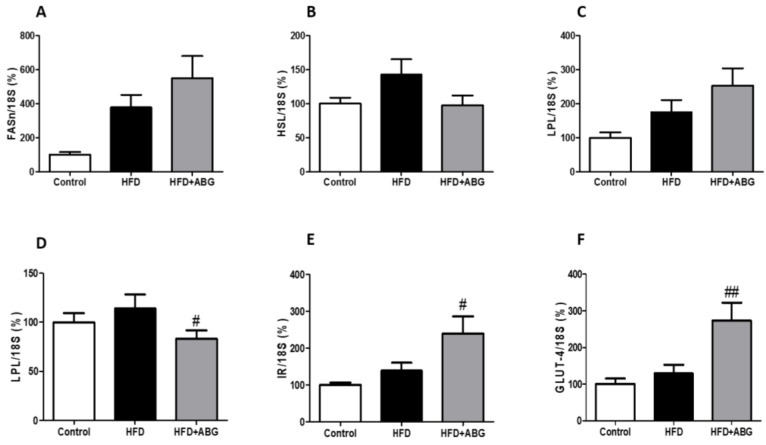
mRNA concentrations of fatty acid synthetase (**A**), hormone-sensitive lipase (**B**), lipoprotein lipase (**C**), peroxisome proliferator-activated receptor gamma (**D**), insulin receptor (**E**) and glucose transporter type 4 (**F**) in brown adipose tissue of rats fed a standard chow (control) or a high-fat diet (HFD)/high-sucrose diet and treated with vehicles or HFD + ABG. Data are represented as mean ± SEM; *n* = 8 samples/group. ^#^ *p* < 0.05 vs. HFD; ^##^ *p* < 0.01 vs. HFD.

**Figure 7:**
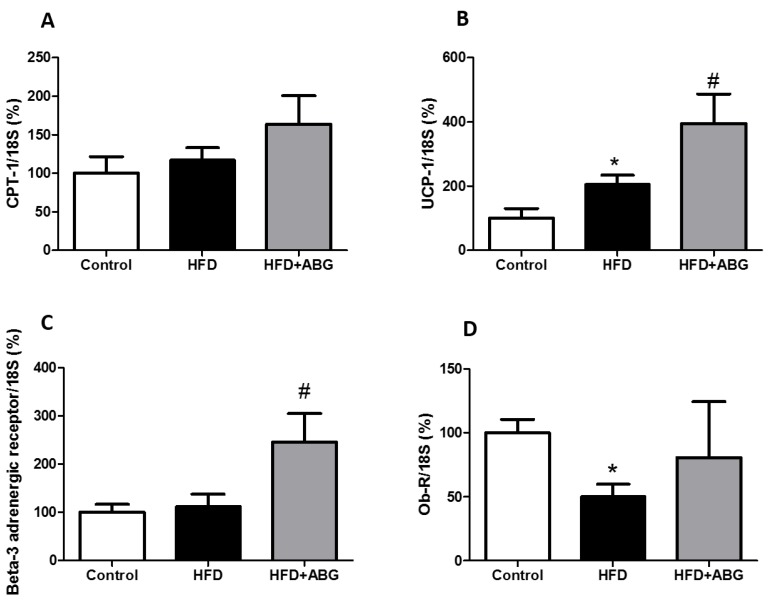
mRNA amounts of carnitine palmitoyltransferase I (**A**), uncoupling protein 1 (**B**), beta-3 adrenergic receptor (**C**) and leptin receptor (**D**) in brown adipose tissue of rats fed a standard chow (control) or a high-fat diet (HFD)/sucrose diet and treated with vehicles or HFD + ABG. Data are represented as mean ± SEM; *n* = 8 samples/group. * *p* < 0.05 vs. control; ^#^ *p* < 0.05 vs. HFD.

In brown adipose tissue, the mRNA levels of FASN, HSL, LPL, PPAR-γ, InsR and GLUT-4 are shown in Figure 6 and the mRNA levels of CPT-1, UCP-1, ObR and β3-ADR are shown in Figure 7. 

No significant differences were found between controls and HFD rats except for an increased gene expression of *UCP-1* (*p* < 0.05; Figure 7B) and decreased ObR mRNA levels in HFD rats compared to controls (*p* < 0.05; Figure 7D). 

ABG treatment did not modify the gene expression of *FASN*, *HSL*, *LPL*, *CPT-1* and *ObR* in brown adipose tissue, but it significantly decreased the gene expression of *PPAR-γ* (*p* < 0.05; Figure 6D) and increased the gene expression of *InsR* (*p* < 0.05; Figure 6E), *GLUT-4* (*p* < 0.01; Figure 6F), *UCP-1* (*p* < 0.05; Figure 7B) and *β-ADR* (*p* < 0.05; Figure 7C).

#### 3.2.9. Vascular Response of Aortic Rings to Potassium Chloride and Acetylcholine

**Figure 8:**
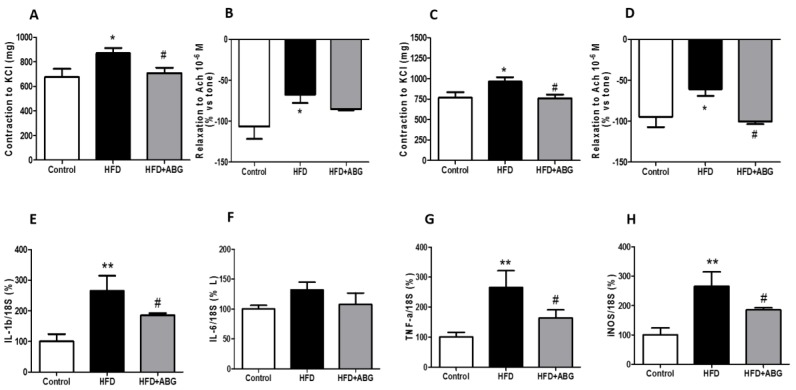
Vascular response of thoracic aortic rings to KCl (100 mM) and acetylcholine (10^−6^ M) in absence (**A**,**B**) or presence (**C**,**D**) of perivascular adipose tissue and mRNA expression of interleukin -1 beta (IL-1β) (**E**), interleukin-6 (IL-6) (**F**), tumor necrosis factor-alpha (TNF-α) (**G**) and inducible nitric oxide synthase (iNOS) (**H**) of rats fed a standard chow (control) or a high-fat diet (HFD)/high-sucrose diet and treated with vehicles or HFD + ABG. Data are represented as mean ± SEM; *n* = 8 samples/group. * *p* < 0.05 vs. control; ** *p* < 0.01 vs. control; ^#^ *p* < 0.05 vs. HFD.

The vascular response of aortic rings denuded or surrounded by PVAT to potassium chloride (100 Mm) and acetylcholine (10^−6^ M) is represented in Figure 8. 

HFD rats showed decreased vasodilation in response to Ach (*p* < 0.05) and increased contraction in response to KCl (*p* < 0.05) both in denuded (Figure 8A,B) and in aorta segments covered with PVAT (Figure 8C,D). 

The relaxing response of aorta segments to Ach was significantly higher in aorta rings from ABG-treated rats compared to non-treated rats only when PVAT was present (*p* < 0.05; Figure 8D). On the contrary, treatment with ABG significantly reduced the contractile response of aorta segments to potassium chloride, regardless of the presence/absence of PVAT (*p* < 0.05; Figure 8A,C).

#### 3.2.10. mRNA levels of IL-1β, IL-6, TNF-α and iNOS in Perivascular Adipose Tissue

No changes were found in the gene expression of *IL-6* among experimental groups (Figure 8F). However, ABG treatment attenuated the obesity-induced increase in the gene expression of *IL-1β* (*p* < 0.01; Figure 8E), *TNF-α* (*p* < 0.05; Figure 8G) and *iNOS* (*p* < 0.01; Figure 8H). 

## 4. Discussion

This manuscript reports the beneficial effects of an ABG extract (ABG10+®) in the metabolic and vascular alterations induced by the consumption of a high-fat/high-sucrose diet in male rats. To our knowledge, this is the first study showing the effects of an ABG extract in the gene expression of proteins and neuropeptides involved in inflammation, fat metabolism and food intake regulation.

Regarding the extract composition, the quantitative results of SAC obtained by HPLC-PAD or HPLC–MS were similar for all ABG10+® samples (around 1.12 mg/g) and in accordance with those obtained by Jung et al. [22]. SAC, together with alliin, was the major components found in ABG10+®. These results were expected as it is reported that during the ageing process, heat accelerates the Maillard reaction, responsible for the brown coloring of the garlic bulbs, and the formation of higher concentrations of organosulphur compounds such as SAC by the enzyme γ-glutamyltransferase [6,20]. This compound is associated with the inhibition of oxidative damage due to its antioxidant capacity [28] and may be related, at least in part, with the beneficial vascular and metabolic effects observed in this study. Indeed, several studies report that both antioxidant nutrients and phytochemicals can provide an advantage in alleviating some of the complications derived from metabolic syndrome [29,30]. In our opinion, SAC and alliin (SAC sulfoxide) might be the major contributors to the beneficial metabolic and vascular effects found in this study, as they are the most abundant components in the ABG10+ extract. In support of this, there are some recent studies reporting the hypoglycemic and hypolipidemic effects of alliin in adult diet-induce-obese (DIO) mice [31], and the protective metabolic effects of SAC in rat pups fed with 20% fructose during the postnatal period [32]. However, we cannot rule out the possibility that other compounds found in the ABG extract, despite being minority, could also play a role in the observed biological effects. 

Our in vivo results show that ABG treatment for one month decreased the mean caloric intake of the animals, due to a significant decrease in the consumption of 5% sucrose solution, with this fact being translated into a lower body weight at the end of the treatment. These results are in agreement with those described by other authors who have reported that ABG treatment decreases body weight in rats fed an HFD [19]. In addition to the decreased body weight, our results also showed a significant decrease in subcutaneous and brown adipose tissues without changes in the weight of epididymal and periaortic visceral adipose tissue depots. On the contrary, it has been previously described that ABG treatment decreases the epididymal visceral adipose tissue depot in male SD rats [19,33] and the periovarian adipose tissue depot in ICR female mice fed an HFD [34]. These contradictory results may be explained by the different compositions and to the different sexes and species used in the different studies (male SD rats vs. female ICR mice). The dosages are also an important factor to be taken into account. In fact, the dosage used in this study and in previous ones [18] are probably too high compared to that advised for humans (around 70 times higher), which may constitute a limitation of the study. Likewise, further studies are required to assess the bioavailability of ABG between rats and humans.

An important finding of our study is that ABG-treated rats showed an improved lipid profile compared to non-treated rats. In this regard, we have found that although ABG treatment did not induce any changes in total cholesterol levels, it significantly decreased serum triglycerides and LDL cholesterol and increased HDL in the serum. These results are in agreement with previous studies in which aged garlic administration attenuated the obesity-induced alterations in the lipid profile both in experimental animals [14,19,21,34] and in humans [22]. However, there is also a recent clinical study in which treatment with aged garlic did not improve the lipid profile in type 2 diabetic patients [35], which possibly suggests that the effectiveness of aged garlic improving the lipid profile may vary depending on different pathological contexts. 

As previously described, ABG treatment not only caused an improvement in the lipid profile of HFD animal, but it also increased the serum levels of adiponectin [36] and decreased the serum concentrations of leptin [14] and insulin [14,37] in HFD rats. However, changes in insulin concentration were not accompanied by a decrease in glycaemia. Likewise, previous studies have reported no effects of ABG-lowering glycaemia in HFD rats [19,37]. Regarding insulin resistance, our results show decreased glycemia in ABG-treated rats 30 minutes after an oral bolus of glucose compared to non-treated rats, pointing out to a slight insulin-sensitizing effect. However, neither HFD- nor HFD + ABG-treated rats recover the basal glycemia 120 minutes after the oral bolus of glucose, which suggests a partial insulin resistance in both experimental groups. In this regard, some authors have reported no effects of aged garlic preventing insulin resistance in type 2 diabetic patients [35], whereas others have reported beneficial effects, increasing insulin sensitivity in HFD rats [37], in db/db mice [21] and in Tsumura Suzuki Obese Diabetes (TSOD) mice [38]. These contradictory results suggest that the insulin-sensitizing effects of ABG may vary depending on the specie and/or the experimental model. 

In order to explain the changes in body weight and improved lipid profile of ABG-treated rats, the mRNA levels of different markers related to energy homeostasis in the hypothalamus and in different adipose tissue depots were analyzed. 

In the hypothalamus, we found an increased gene expression of the anorexigenic neuropeptide *POMC* and a decreased gene expression of the leptin receptor *ObR* in ABG-treated rats compared to non-treated rats. The decreased *ObR* gene expression may be related to the decreased circulating leptin and the increased POMC mRNA concentrations may be responsible, at least in part, for the decreased caloric intake of ABG-treated rats, which was mainly due to the decreased intake of water + 5% sucrose. In our opinion, this is relevant and may have a potential interest for the pharmaceutical and the food industries that have a growing interest for finding a product that selectively inhibits the consumption of sugars. Indeed, a recent study has postulated that in a high-fat/high-carbohydrate diet, sugars, instead of lipids, are the ones responsible for the induction of hypothalamic inflammation, which affects, among others, the POMC neurons, with this fact very likely leading to neuronal dysfunction in the control of energy metabolism [39]. As the gene expression of *TNF-α* and *IL-1β* was significantly upregulated in HFD rats compared to that in controls but not in ABG-treated rats, it is possible that the lower hypothalamic inflammation in these rats is associated with a lower neuronal damage in POMC neurons that may justify the increased POMC mRNA levels and the decreased consumption of water + 5% sugar. In agreement with our results, it has been recently reported that treatments like proanthocyanidins, which reduce obesity-induced hypothalamic inflammation and leptin resistance, are associated with decreased caloric intake and increased gene expression of *POMC* in the hypothalamus [40]. 

In epididymal adipose tissue, no changes were found, neither in the weight nor in the gene expression of proteins involved in lipid metabolism, except for a marked increase in the mRNA levels of the β3-ADR. This is relevant as this receptor is reported to play a key role in insulin sensitivity [41], and to mediate leptin-induced lipolytic effects in adipose tissue through the activation of the sympathetic nervous system [42,43]. In fact, it is reported that increased adiposity in an obesity context is the result of a reduction of beta-adrenergic agonist-induced lipolysis, which could be due to both losses of beta-1 and beta-3 adrenoceptor numbers, or to alterations of their coupling to adenylate cyclase through the guanine nucleotide regulatory protein [44]. Particularly, in diet-induced obesity in rats, it is reported that increased adiposity is associated with both decreased gene expression of β-adrenoceptors and impaired activation of the β-adrenoceptor-cAMP-PKA-HSL pathway in adipose tissue [45]. Furthermore, treatments that reduce adiposity are sometimes associated with an increased gene expression of β3-adrenoceptor in adipose tissue [46,47]. 

Despite epididymal adipose tissue, significant changes were found in the weight and in the gene expression of different markers, both in subcutaneous and in brown adipose tissue, in response to ABG treatment. The decreased weight of subcutaneous adipose tissue is most likely related, not only to the reduced caloric intake, but also to the decreased LPL mRNA levels, since this insulin-dependent enzyme is involved in triglycerides uptake and fat deposition [48]. However, the mRNA levels of both ObR and the lipolytic enzyme HSL were also decreased, which possibly indicates that the decrease in the weight of this tissue is due to a decrease in fat deposition rather than an increase in lipolysis. In addition, ABG may also affect adipogenesis as the gene expression of *PPAR-γ* was significantly decreased, not only in subcutaneous, but also in brown adipose tissue of ABG-treated rats. Likewise, it has been previously reported that ABG inhibits adipocyte differentiation and adipogenesis in 3T3-L1 preadipocytes in vitro [49]. 

In brown adipose tissue, we found a significant upregulation of β3-ADR, InsR and GLUT-4, which may suggest improved insulin sensitivity in this tissue [41]. Likewise, it has been previously described that treatment with aged garlic improves insulin sensitivity through the upregulation of the phosphorylated AMP-activated protein kinase (AMPK) in the adipose tissue, liver and muscle of TSOD mice [38]. In addition, we also found an upregulation in the mRNA levels of UCP-1 and a downregulation in the gene expression of *LPL*, which is probably related with a decreased lipid uptake and/or increased thermogenesis in these animals that might justify the decreased brown adipose tissue (BAT) weight. Similar effects had already been reported in mice treated with raw garlic [50], but to our knowledge, this is the first study reporting these effects with a commercial ABG extract. 

Our results oppose to those described by Ahmadi et al., [51] who reported that an extract of aged garlic supplemented with vitamin B12, folic acid, vitamin B6 and L-arginine for 12 months increased the ratio between brown and white adipose tissue in the human epicardium. The opposing results between these two studies may be explained by differences in the extract composition (pure ABG extract vs. aged garlic supplemented with vitamins and aminoacids), in the different brown adipose depots analyzed (interscapular vs. epicardial) and in the different species used (rats vs. humans). However, the decreased BAT weight in ABG-treated rats was not completely unexpected, since other studies in the literature report that in an obesity context, BAT weight increases [52,53]. Furthermore, certain treatments that exert a decrease in body weight also reduce both BAT weight and BAT inflammation [52].

Finally, previous studies have reported that diet-induced obesity is associated with altered vascular function, including an increased vascular contraction in response to KCl and a decreased vascular relaxation in response to acetylcholine [54]. Our results showed that ABG treatment significantly decreased the contraction of aortic rings in response to KCl and increased the relaxation of aorta segments in response to acetylcholine pointing to a beneficial effect on vascular function. The increased relaxation in response to acetylcholine reveals an improvement in obesity-induced endothelial dysfunction. However, this beneficial effect was only evident in aorta segments surrounded by PVAT, which may be related, at least in part, to the decreased gene expression of pro-inflammatory markers in PVAT [55]. On the contrary, vascular contraction in response to KCl was decreased in aorta segments from ABG-treated rats regardless of the presence/absence of PVAT, which points out that the endothelium may result more affected by the presence of inflamed PVAT than the vascular wall. Further experiments are required to analyze the mechanisms of decreased contraction in response to KCl in ABG10-treated rats, although it may be possibly due, among other causes, to a reduction in the thickness of the smooth muscle layer, altered Ca^2+^ dynamics, and/or improved NO bioavailability. 

In conclusion, ABG administration decreases caloric intake and body weight in diet-induced obese rats through modifications in the gene expression of different markers related to insulin sensitivity and fat metabolism in adipose tissue and through an increased expression of anorexigenic neuropeptides in the hypothalamus. Furthermore, ABG treatment improves vascular function possibly by an improvement of the pro-inflammatory profile of PVAT. As ABG10+ treatment significantly reduced body weight gain in HFD-fed rats, the improved metabolic and vascular profiles of these animals may be the result of the decrease in body weight.

The results derived from this work are relevant as, to our knowledge, they report for the first time the beneficial vascular and metabolic effects of ABG in an obesity context. These findings may help in the search of new natural ant obesity therapies as an alternative to the traditional pharmacological treatments that, in general, are related to higher side effects. 

## Figures and Tables

**Table 1:** Identification and quantification of organosulphur components by HPLC–MS in ABG10+®. For each compound the concentration, electrospray ionization (ESI) and molecular mass (MM) are shown.

T_R_ (min)	Compound	ESI + (*m/z*)	MM (Da)	Chemical Structure	Concentration (mg/g)
3.9	γ-L-glutamyl-S-methyl-L-cysteine (GSMC)	265	264	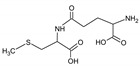	0.00 ± 0.00
4.5	(+)-S-methyl-L-cysteine sulfoxide (methiin)	104	103	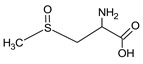	0.00 ± 0.00
4.9	(+)-S-allyl-L-cysteine sulfoxide (alliin)	178	177	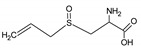	0.03 ± 0.00
5.2	(+)-S-(*trans*-1-propenyl)-L-cysteine sulfoxide (*iso*-alliin)	178	177	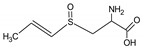	0.01 ± 0.00
7.41	S-allyl cysteine (SAC)	162	161	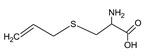	1.15 ± 0.02
8.8	*iso*-S-allyl-L-cystein (*iso*-SAC)	162	161		0.04 ± 0.00
10.9	γ-L-glutamyl-S-allyl-L-cysteine (GSAC)	291	290	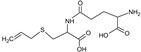	0.00 ± 0.00
11.6	γ-L-glutamyl-S-(*trans*-1-propenyl)-L-cysteine (GSPC)	291	290	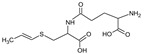	0.00 ± 0.00

**Table 2:** Organ weights, lipid profile and hormone concentrations in the serum of rats fed a standard chow (control) or a high-fat diet (HFD)/high-sucrose diet and treated with vehicles or HFD + ABG.

	Control	HFD	HFD + ABG
Heart (g)	1.58 ± 0.03	1.72 ±0.02	1.85 ± 0.07
Kidneys (g)	2.33 ± 0.08	2.36 ± 0.10	2.30 ± 0.05
Adrenal glands (mg)	48.5 ± 1.6	48.3 ± 1.7	51.6 ± 2.2
Spleen (g)	0.84 ± 0.04	0.82 ± 0.05	0.88 ± 0.04
Liver (g)	9.79 ± 0.17	10.32 ± 0.44	10.27 ± 0.47
Epidydimal visceral fat (g)	5.31 ± 0.28	10.24 ± 0.97 ***	10.00 ± 0.75
Lumbar subcutaneous fat (g)	2.41 ±0.13	4.62 ± 0.33 ***	3.84 ± 0.21 ^#^
Brown adipose tissue (g)	0.67 ± 0.06	1.20 ± 0.05 ***	0.88 ± 0.03 ^###^
Periaortic adipose tissue (g)	0.057 ± 0.006	0.113 ± 0.015 **	0.111 ± 0.016
Soleus (g)	0.18 ± 0.04	0.20 ± 0.07	0.21 ± 0.01
Gastrocnemius (g)	2.28 ± 0.05	2.52 ± 0.07	2.50 ± 0.07
Glycemia (mg/dL)	67 ± 2	63.5 ± 2	63.6 ± 3
Total Cholesterol (mg/dL)	161 ± 8	187 ± 10 *	180 ± 9
LDL-cholesterol (mg/dL)	86 ± 3	94 ± 3 *	79 ± 6 ^#^
HDL-cholesterol (mg/dL)	111 ± 4	74 ± 6 **	108 ± 7 ^##^
Tryglycerides (mg/dL)	105 ± 9	162 ± 16 **	127 ± 12 ^#^
Insulin (ng/mL)	3.5 ± 0.5	5.5 ± 0.6 *	3.5 ± 0.4 ^#^
Leptin (ng/mL)	6.4 ± 3.2	19.4 ± 3 **	9.4 ± 1.6 ^#^
Adiponectin (mg/dL)	104 ± 5	109 ± 9	151 ± 13 ^#^

Data are represented as mean value ± SEM; *n* = 8–12 samples/group. * *p* < 0.05 vs. control; ** *p* < 0.01 vs. control; *** *p* < 0.001 vs. control; ^#^ *p* < 0.05 vs. HFD; ^##^ *p* < 0.01 vs. HFD; ^###^ *p* < 0.001 vs. HFD. Organ and tissue weights were compared after removing the influence of body weight by ANCOVA.

## References

[1] Ferrante A.W. (2013). Macrophages, fat, and the emergence of immunometabolism. J. Clin. Investig..

[2] Pirola L., Ferraz J.C. (2017). Role of pro- and anti-inflammatory phenomena in the physiopathology of type 2 diabetes and obesity. World J. Biol. Chem..

[3] Kang J.G., Park C.Y. (2012). Anti-Obesity Drugs: A Review about Their Effects and Safety. Diabetes Metab. J..

[4] Kimura S., Tung Y.C., Pan M.H., Su N.W., Lai Y.J., Cheng K.C. (2017). Black garlic: A critical review of its production, bioactivity, and application. J. Food Drug Anal..

[5] Ryu J.H., Kang D. (2017). Physicochemical Properties, Biological Activity, Health Benefits, and General Limitations of Aged Black Garlic: A Review. Molecules.

[6] Bae S.E., Cho S.Y., Won Y.D., Lee S.H., Park H.J. (2012). A comparative study of the different analytical methods for analysis of S-allyl cysteine in black garlic by HPLC. LWT-Food Sci. Technol..

[7] Lindenmeier M., Faist V., Hofmann T. (2002). Structural and functional characterization of pronyl-lysine, a novel protein modification in bread crust melanoidins showing in vitro antioxidative and phase I/II enzyme modulating activity. J. Agric. Food Chem..

[8] Moreira A.S., Nunes F.M., Domingues M.R., Coimbra M.A. (2012). Coffee melanoidins: Structures, mechanisms of formation and potential health impacts. Food Funct..

[9] Borrelli R.C., Visconti A., Mennella C., Anese M., Fogliano V. (2002). Chemical characterization and antioxidant properties of coffee melanoidins. J. Agric. Food Chem..

[10] Wang Y., Ho C.T. (2009). Polyphenolic chemistry of tea and coffee: A century of progress. J. Agric. Food Chem..

[11] Kim D.G., Kang M.J., Hong S.S., Choi Y.H., Shin J.H. (2017). Antiinflammatory Effects of Functionally Active Compounds Isolated from Aged Black Garlic. Phytother. Res..

[12] Nillert N., Pannangrong W., Welbat J.U., Chaijaroonkhanarak W., Sripanidkulchai K., Sripanidkulchai B. (2017). Neuroprotective Effects of Aged Garlic Extract on Cognitive Dysfunction and Neuroinflammation Induced by beta-Amyloid in Rats. Nutrients.

[13] Jikihara H., Qi G., Nozoe K., Hirokawa M., Sato H., Sugihara Y., Shimamoto F. (2015). Aged garlic extract inhibits 1,2-dimethylhydrazine-induced colon tumor development by suppressing cell proliferation. Oncol. Rep..

[14] Perez-Torres I., Torres-Narvaez J.C., Pedraza-Chaverri J., Rubio-Ruiz M.E., Diaz-Diaz E., Del Valle-Mondragon L., Martinez-Memije R., Varela Lopez E., Guarner-Lans V. (2016). Effect of the Aged Garlic Extract on Cardiovascular Function in Metabolic Syndrome Rats. Molecules.

[15] Baluchnejadmojarad T., Kiasalari Z., Afshin-Majd S., Ghasemi Z., Roghani M. (2017). S-allyl cysteine ameliorates cognitive deficits in streptozotocin-diabetic rats via suppression of oxidative stress, inflammation, and acetylcholinesterase. Eur. J. Pharmacol..

[16] Yoo J.M., Sok D.E., Kim M.R. (2014). Anti-allergic action of aged black garlic extract in RBL-2H3 cells and passive cutaneous anaphylaxis reaction in mice. J. Med. Food..

[17] Garcia-Villalón A.L., Amor S., Monge L., Fernández N., Prodanov M., Muñoz M., Inarejos-García A.M., Granado M. (2016). In vitro studies of an aged black garlic extract enriched in S-allylcysteine and polyphenols with cardioprotective effects. J. Funct. Foods.

[18] Shin J.H., Lee C.W., Oh S.J., Yun J., Kang M.R., Han S.B., Park H., Jung J.C., Chung Y.H., Kang J.S. (2014). Hepatoprotective effect of aged black garlic extract in rodents. Toxicol. Res..

[19] Ha A.W., Ying T., Kim W.K. (2015). The effects of black garlic (Allium satvium) extracts on lipid metabolism in rats fed a high fat diet. Nutr. Res. Pract..

[20] Kim J.H., Yu S.H., Cho Y.J., Pan J.H., Cho H.T., Kim J.H., Bong H., Lee Y., Chang M.H., Jeong Y.J. (2017). Preparation of S-Allylcysteine-Enriched Black Garlic Juice and Its Antidiabetic Effects in Streptozotocin-Induced Insulin-Deficient Mice. J. Agric. Food Chem..

[21] Lee Y.M., Gweon O.C., Seo Y.J., Im J., Kang M.J., Kim M.J., Kim J.I. (2009). Antioxidant effect of garlic and aged black garlic in animal model of type 2 diabetes mellitus. Nutr. Res. Pract..

[22] Jung E.S., Park S.H., Choi E.K., Ryu B.H., Park B.H., Kim D.S., Kim Y.G., Chae S.W. (2014). Reduction of blood lipid parameters by a 12-wk supplementation of aged black garlic: A randomized controlled trial. Nutrition.

[23] Kang O.J. (2016). Evaluation of Melanoidins Formed from Black Garlic after Different Thermal Processing Steps. Prev. Nutr. Food Sci..

[24] Haffner S.M., Stern M.P., Hazuda H.P., Pugh J.A., Patterson J.K. (1986). Hyperinsulinemia in a population at high risk for non-insulin-dependent diabetes mellitus. N. Engl. J. Med..

[25] Roza N.A., Possignolo L.F., Palanch A.C., Gontijo J.A. (2016). Effect of long-term high-fat diet intake on peripheral insulin sensibility, blood pressure, and renal function in female rats. Food Nutr. Res..

[26] Chomczynski P. (1993). A reagent for the single-step simultaneous isolation of RNA, DNA and proteins from cell and tissue samples. Biotechniques.

[27] Livak K.J., Schmittgen T.D. (2001). Analysis of relative gene expression data using real-time quantitative PCR and the 2(-Delta Delta C(T)) Method. Methods.

[28] Colin-Gonzalez A.L., Santana R.A., Silva-Islas C.A., Chanez-Cardenas M.E., Santamaria A., Maldonado P.D. (2012). The antioxidant mechanisms underlying the aged garlic extract- and S-allylcysteine-induced protection. Oxid. Med. Cell Longev..

[29] Ilkun O., Boudina S. (2013). Cardiac dysfunction and oxidative stress in the metabolic syndrome: An update on antioxidant therapies. Curr. Pharm. Des..

[30] Xia X., Weng J. (2010). Targeting metabolic syndrome: Candidate natural agents. J. Diabetes.

[31] Zhai B., Zhang C., Sheng Y., Zhao C., He X., Xu W., Huang K., Luo Y. (2018). Hypoglycemic and hypolipidemic effect of S-allyl-cysteine sulfoxide (alliin) in DIO mice. Sci. Rep..

[32] Lembede B.W., Erlwanger K.H., Nkomozepi P., Chivandi E. (2018). Effect of neonatal orally administered S-allyl cysteine in high-fructose diet fed Wistar rats. J. Dev. Orig. Health Dis..

[33] Seo D.Y., Lee S., Figueroa A., Kwak Y.S., Kim N., Rhee B.D., Ko K.S., Bang H.S., Baek Y.H., Han J. (2012). Aged garlic extract enhances exercise-mediated improvement of metabolic parameters in high fat diet-induced obese rats. Nutr. Res. Pract..

[34] Jung Y.M., Lee S.H., Lee D.S., You M.J., Chung I.K., Cheon W.H., Kwon Y.S., Lee Y.J., Ku S.K. (2011). Fermented garlic protects diabetic, obese mice when fed a high-fat diet by antioxidant effects. Nutr. Res..

[35] Atkin M., Laight D., Cummings M.H. (2016). The effects of garlic extract upon endothelial function, vascular inflammation, oxidative stress and insulin resistance in adults with type 2 diabetes at high cardiovascular risk. A pilot double blind randomized placebo controlled trial. J. Diabetes Complic..

[36] Gomez-Arbelaez D., Lahera V., Oubina P., Valero-Munoz M., de Las Heras N., Rodriguez Y., Garcia R.G., Camacho P.A., Lopez-Jaramillo P. (2013). Aged garlic extract improves adiponectin levels in subjects with metabolic syndrome: A double-blind, placebo-controlled, randomized, crossover study. Mediat. Inflamm..

[37] Seo D.Y., Kwak H.B., Lee S.R., Cho Y.S., Song I.S., Kim N., Bang H.S., Rhee B.D., Ko K.S., Park B.J. (2014). Effects of aged garlic extract and endurance exercise on skeletal muscle FNDC-5 and circulating irisin in high-fat-diet rat models. Nutr. Res. Pract..

[38] Miki S., Inokuma K.I., Takashima M., Nishida M., Sasaki Y., Ushijima M., Suzuki J.I., Morihara N. (2017). Aged garlic extract suppresses the increase of plasma glycated albumin level and enhances the AMP-activated protein kinase in adipose tissue in TSOD mice. Mol. Nutr. Food Res..

[39] Gao Y., Bielohuby M., Fleming T., Grabner G.F., Foppen E., Bernhard W., Guzman-Ruiz M., Layritz C., Legutko B., Zinser E. (2017). Dietary sugars, not lipids, drive hypothalamic inflammation. Mol. Metab..

[40] Ibars M., Ardid-Ruiz A., Suarez M., Muguerza B., Blade C., Aragones G. (2017). Proanthocyanidins potentiate hypothalamic leptin/STAT3 signalling and Pomc gene expression in rats with diet-induced obesity. Int. J. Obes..

[41] Ishino S., Sugita T., Kondo Y., Okai M., Tsuchimori K., Watanabe M., Mori I., Hosoya M., Horiguchi T., Kamiguchi H. (2017). Glucose uptake of the muscle and adipose tissues in diabetes and obesity disease models: Evaluation of insulin and beta3-adrenergic receptor agonist effects by (18)F-FDG. Ann. Nucl. Med..

[42] Mark A.L., Rahmouni K., Correia M., Haynes W.G. (2003). A leptin-sympathetic-leptin feedback loop: Potential implications for regulation of arterial pressure and body fat. Acta Physiol. Scand..

[43] Zeng W., Pirzgalska R.M., Pereira M.M., Kubasova N., Barateiro A., Seixas E., Lu Y.H., Kozlova A., Voss H., Martins G.G. (2015). Sympathetic neuro-adipose connections mediate leptin-driven lipolysis. Cell.

[44] Portillo M.P., Simon E., Garcia-Calonge M.A., Del Barrio A.S. (1999). Effect of high-fat diet on lypolisis in isolated adipocytes from visceral and subcutaneous WAT. Eur. J. Nutr..

[45] Ding L., Zhang F., Zhao M.X., Ren X.S., Chen Q., Li Y.H., Kang Y.M., Zhu G.Q. (2016). Reduced lipolysis response to adipose afferent reflex involved in impaired activation of adrenoceptor-cAMP-PKA-hormone sensitive lipase pathway in obesity. Sci. Rep..

[46] Fu J., Zeng C., Zeng Z., Wang B., Wen X., Yu P., Gong D. (2016). Cinnamomum camphora Seed Kernel Oil Improves Lipid Metabolism and Enhances beta3-Adrenergic Receptor Expression in Diet-Induced Obese Rats. Lipids.

[47] Yang L., Lu K., Wen X.Y., Liu H., Chen A.P., Xu M.W., Zhang H., Yu J. (2012). Jueming Prescription reduces body weight by increasing the mRNA expressions of beta3-adrenergic receptor and uncoupling protein-2 in adipose tissue of diet-induced obese rats. Chin. J. Integr. Med..

[48] Mead J.R., Irvine S.A., Ramji D.P. (2002). Lipoprotein lipase: Structure, function, regulation, and role in disease. J. Mol. Med..

[49] Park J.-A. (2011). Inhibition of Adipocyte Differentiation and Adipogenesis by Aged Black Garlic Extracts in 3T3-L1 Preadipocytes. J. Life Sci..

[50] Kim M.J., Kim H.K. (2011). Effect of garlic on high fat induced obesity. Acta Biol. Hung.

[51] Ahmadi N., Nabavi V., Hajsadeghi F., Zeb I., Flores F., Ebrahimi R., Budoff M. (2013). Aged garlic extract with supplement is associated with increase in brown adipose, decrease in white adipose tissue and predict lack of progression in coronary atherosclerosis. Int. J. Cardiol..

[52] Bao B., Chen Y.G., Zhang L., Na Xu Y.L., Wang X., Liu J., Qu W. (2013). Momordica charantia (Bitter Melon) reduces obesity-associated macrophage and mast cell infiltration as well as inflammatory cytokine expression in adipose tissues. PLoS ONE.

[53] Sampey B.P., Vanhoose A.M., Winfield H.M., Freemerman A.J., Muehlbauer M.J., Fueger P.T., Newgard C.B., Makowski L. (2011). Cafeteria diet is a robust model of human metabolic syndrome with liver and adipose inflammation: Comparison to high-fat diet. Obesity.

[54] Alarcon G., Roco J., Medina M., Medina A., Peral M., Jerez S. (2018). High fat diet-induced metabolically obese and normal weight rabbit model shows early vascular dysfunction: Mechanisms involved. Int. J. Obes..

[55] Nosalski R., Guzik T.J. (2017). Perivascular adipose tissue inflammation in vascular disease. Br. J. Pharmacol..

